# Face mask use during the COVID-19 pandemic: how risk perception, experience with COVID-19, and attitude towards government interact with country-wide policy stringency

**DOI:** 10.1186/s12889-022-13632-9

**Published:** 2022-08-26

**Authors:** Annelot Wismans, Peter van der Zwan, Karl Wennberg, Ingmar Franken, Jinia Mukerjee, Rui Baptista, Jorge Barrientos Marín, Andrew Burke, Marcus Dejardin, Frank Janssen, Srebrenka Letina, José María Millán, Enrico Santarelli, Olivier Torrès, Roy Thurik

**Affiliations:** 1 Department of Applied Economics, Erasmus School of Economics, Erasmus University Rotterdam, PO Box 1738, 3000DR Rotterdam, The Netherlands; 2 The Erasmus University Rotterdam Institute for Behavior and Biology (EURIBEB), P.O. Box 1738, 3000DR Rotterdam, The Netherlands; 3 Department of Business Studies, Institute of Tax Law and Economics, Leiden Law School, Leiden University, 2311ES Leiden, The Netherlands; 4 Institute for Analytical Sociology, Linköping University, SE-601 74 Norrköping, Sweden; 5 Stockholm School of Economics, PO Box 6501, SE-113 83 Stockholm, Sweden; 6 Department of Psychology, Education & Child Studies, Erasmus University Rotterdam, PO Box 1738, 3000DR Rotterdam, The Netherlands; 7 Montpellier Business School, CEDEX 4, 2300 Avenue des Moulins, 34080 Montpellier, France; 8 CEG-IST, Instituto Superior Técnico, University of Lisbon, 1049-001 Lisbon, Portugal; 9 Department of Economics, University of Antioquia, PO Box 1228, Calle 70 52-21, Medellín, Colombia; 10 Trinity Business School, Trinity College Dublin, Dublin 2, D02 H308 Ireland; 11 Université catholique de Louvain, B-1348 Louvain-la-Neuve, Belgium; 12 Université de Namur, B-5000 Namur, Belgium; 13 University of Glasgow, Glasgow, G12 8QQ UK; 14 Department of Economics, University of Huelva, 21004 Huelva, Spain; 15 Department of Economics, University of Bologna, 40126 Bologna, Italy; 16 University of Montpellier, 34000 Montpellier, France

**Keywords:** Face mask, Compliance, COVID-19, Students, Multilevel analysis, Policy stringency

## Abstract

**Background:**

During the 2020 COVID-19 pandemic, governments imposed numerous regulations to protect public health, particularly the (mandatory) use of face masks. However, the appropriateness and effectiveness of face mask regulations have been widely discussed, as is apparent from the divergent measures taken across and within countries over time, including mandating, recommending, and discouraging their use. In this study, we analyse how country-level policy stringency and individual-level predictors associate with face mask use during the early stages of the global COVID-19 pandemic.

**Method:**

First, we study how (self and other-related) risk perception, (direct and indirect) experience with COVID-19, attitude towards government and policy stringency shape face mask use. Second, we study whether there is an interaction between policy stringency and the individual-level variables. We conduct multilevel analyses exploiting variation in face mask regulations across countries and using data from approximately 7000 students collected in the beginning of the pandemic (weeks 17 through 19, 2020).

**Results:**

We show that policy stringency is strongly positively associated with face mask use. We find a positive association between self-related risk perception and mask use, but no relationship of mask use with experience with COVID-19 and attitudes towards government. However, in the interaction analyses, we find that government trust and perceived clarity of communication moderate the link between stringency and mask use, with positive government perceptions relating to higher use in countries with regulations and to lower use in countries without regulations.

**Conclusions:**

We highlight that those countries that aim for widespread use of face masks should set strict measures, stress self-related risks of COVID-19, and use clear communication.

**Supplementary Information:**

The online version contains supplementary material available at 10.1186/s12889-022-13632-9.

## Introduction

Mandated face mask use has been one of the most contentious topics during the 2020 COVID-19 pandemic. During the early phase of the pandemic, positions on general mandated face mask use were highly divergent across countries and subject to change within countries [1, 2]. Several countries discouraged the use of face masks due to a lack of evidence of its effectiveness, to preserve limited supplies for health care and due to concerns about risk compensation in the form of lowering compliance with other measures [1, 3, 4]. In response to changes in advice from the WHO and with more studies proving the effectiveness of masks [5–9], face mask regulations became more uniform and accepted during later phases of the COVID-19 pandemic. With reoccurring infection outbreaks due to low vaccination rates, but also despite high vaccination rates, for the immediate future face masks may remain to be a cheap, non-invasive, and prudent intervention. In this study, we focus on the initial phase of the pandemic when regulations were divergent. We study the importance of country-level policy stringency, individual-level factors, and their interaction for the use of face masks. Specifically, we study individual attitude towards government, risk perception, and experience with COVID-19. Studying whether these individual-level variables relate differently to face mask use across different stringency contexts is important, especially now that in later phases of the pandemic countries are constantly changing the stringency of measures reacting to peaks and troughs in infection numbers.

Studies have shown that differences in policy stringency across countries and even regions strongly affected the uptake of measures taken to lower the spread of COVID-19, specifically the use of face masks [10–12]. Policy-induced changes result both from a general tendency to obey to authority [13, 14] and from the signal that the enforced behavior is deemed appropriate, reinforcing, or refining a social norm and creating social meaning [15, 16]. Due to regulations, wearing a face mask may have a different social meaning in different countries: from being paranoid or being a person at risk in countries without regulations to being a ‘good citizen’ or abiding by a social contract in countries with regulations. In a large German study, mask-wearing increased rapidly when made mandatory and those wearing masks saw each other as more positive and prosocial, while those not wearing masks were socially “punished”, indicating that regulations imposed a social contract [11]. Moreover, seeing others wearing a mask, a so-called descriptive norm, was found to be a strong determinant of mask use [17]. However, even without policies in place, the outbreak of COVID-19 resulted in voluntary engagement in protective behaviors, like staying at home [18] and mask-wearing [19, 20].

While government policy is effective in changing behavior, individuals’ perception of government is equally important, as individuals with lower trust are found to have a lower willingness to defer to decisions made by government [21, 22]. In the context of pandemics, trust in government has been related to social distancing compliance [23], quarantine adherence [24], acceptance of vaccination [25] and face mask use [10]. Of additional importance is the clarity of communication of authorities, as limited health literacy is associated with poorer health and medication nonadherence [26, 27]. It is crucial that communication be clear and unambiguous. A UK study showed that guidance on social distancing and isolation during the COVID-19 pandemic was unclear, and ‘mixed messages’ were being spread [28]. Research has also noted the prevalence of biased, erroneous, and distortive information regarding COVID-19 and various protective behaviors [29, 30]. Positive perceptions about clarity and consistency of information are related to increased compliance with recommended behaviors [31]. Hence, both trust in government and perceived clarity of communication are expected to strengthen compliance with face mask regulations.

Additionally, multiple studies have underlined the importance of risk perception for compliance with COVID-19 measures [32, 33]. The widely used Health Belief Model depicts health behaviors as driven by individuals’ risk perception of susceptibility and severity of a disease [34]. Not only perceived risk for oneself, but also social risk perception – the perceived risk for those in one’s environment – plays a role in compliance [35]. Perceptions of the social risk of COVID-19 have been related to engaging in protective measures [36, 37]. Relatedly, studies show that antisocial personality traits are linked to lower compliance with regulations [38–40]. In the decision to wear a face mask, the perceived risk of COVID-19 for others could be more important for younger people, who may believe themselves to be less at risk of negative health consequences due to a COVID-19 infection. Asri et al. [41] showed that older people were motivated by self-regarding risk preferences to wear a mask, while younger people were also motivated by other-regarding concerns. In general, both higher self-related and other-related risk perception is expected to have a positive association with mask usage.

Finally, experience is also important for shaping attitudes, beliefs and consequently behavior [42–45], with a distinction being made between direct (personal) experience and indirect experience (of others) [44]. Experience with a disease can both stimulate and discourage preventive behaviors. Shahrabani and Benzion [46] showed that vaccination was perceived less beneficial after influenza-infection. Though, knowing others that suffered from a disease has been positively associated with preventive health behavior [47–49]. Related to face mask use during the pandemic, Cherry et al. [50] showed that testing negative for COVID-19 is associated with increased face mask use support, while testing positive has no effect and in some cases even reduced face mask use support. The latter could be explained by the fact that people may believe that they are immune or less at risk for COVID-19 after infection. Moreover, knowing someone that was infected with COVID-19 is positively related to supporting face mask use and engaging in preventive measures [12, 50, 51], possibly because this increases the saliency of COVID-19 and therefore the perceived need for mask use. Consequently, we expect that direct experience with COVID-19 is associated with lower face mask use, while indirect experience with COVID-19 is associated with higher face mask use.

Studies have shown that relationships between individual-level factors and preventive behavior may be dependent on the context, such as policy stringency. In the case of mobility reduction, it was shown that the effect of policy stringency was more pronounced in high-trust regions relative to low-trust regions [10]. Also, Pak et al. [52] found that individual government trust and perception of government truthfulness increased the predicted compliance as policy stringency increases. In countries without any regulations on mask use, government trust and perceived clarity of communication could even negatively associate with face mask usage, as governments do not actively recommend the behavior. In line with previous studies, we therefore expect that individual attitude towards government positively moderates the association between policy stringency and face mask use.

There are no studies to date looking at the interaction between risk perception or experience and policy stringency. As policy becomes more stringent, it is possible that behavior is more uniformly changed, and social norms become so strong thereby limiting the association of individual differences with face mask use. In situations without regulations, there is less structure and more ambiguity on what behavior to perform, consequently individual differences may play a larger role in behavior. This reasoning is in line with the ‘strong situation hypothesis’, stating that in strong situations – such as nationwide lockdowns – there is a limited range of appropriate behavior, thereby constraining the range of behavioral variability. While the strong situation hypothesis focuses on the reduced influence of personality traits and has been debated [53–56], it is likely that in a context of more stringent regulations attitudes, like risk perception and experience, are less strongly associated with behavior. During the pandemic, Götz et al. (2021) found partial support for the interaction between personality and stringency, with certain traits having weaker effects on sheltering-in-place when policies became stricter. Therefore, we expect that the association between risk perception and experience on the one hand and face mask use on the other hand may differ across different policy stringency contexts.

In this study we will analyze how macrolevel policies and individual-level factors independently and jointly associate with face mask use during the early stages of the global COVID-19 pandemic when regulations on face mask use were divergent. We use data from a large sample of approximately 7000 university students from ten countries (Belgium, Colombia, France, India, Ireland, Italy, the Netherlands, Portugal, Spain, Sweden), collected between 23rd April-12th of May 2020, as part of the Erasmus University Rotterdam International COVID-19 Students Survey [38, 57, 58]. First, we study how (self-related and other-related) risk perception, (direct and indirect) experience with COVID-19, attitude towards government and policy stringency independently shape face mask use. Second, we study whether the association between individual-level factors and face mask use differs across countries with different policy stringency by conducting moderation analyses. The cross-country dataset is analyzed using multilevel regression analyses. The stringency of face mask regulations is captured by using objective data on regulations on face masks in each country [59].

Compared to most of the literature on face mask use, our paper takes a holistic approach by studying how factors that have been previously found to be important for face mask use work out in the context of different regulations (e.g., countries with different face mask policies). Moreover, we are the first to study whether policy stringency moderates the association of perceived clarity of government communication, risk perception and experience with COVID-19 with face mask usage.

## Materials and methods

### Sample

We use data from the first wave of the Erasmus University Rotterdam International COVID-19 Student Survey [38, 57, 58]. The dataset consists of survey data from a large sample of university students from multiple countries. The data were collected during 13 consecutive days in the initial phase of the 2020 COVID-19 pandemic (weeks 17-19, 2020). The survey received approval from the Internal Review Board of the Erasmus University Rotterdam before initiation (ESE IRB-NE Application 2020–05).

The survey was shared with students in Belgium, Colombia, France, India, Ireland, Italy, the Netherlands, Portugal, Spain, and Sweden, primarily using university e-mail addresses and online university platforms. Previous studies have already used this dataset [38, 57, 58]. The survey was completed online using survey software from Qualtrics. Participation was voluntary, and an informed consent form was provided upon the start of the survey. The survey could be completed in four languages: English, Dutch, French, and Spanish. All translations were made by two native speakers.

In total, the sample consists of 7403 students from ten countries. After calculating Little’s MCAR (*X*^*2*^ = 45.76, *p* = .13), we conclude that data are missing completely at random and use listwise deletion. Due to excluding missing data and restricting our sample to students between 17 and 35 years old, the final dataset used for the analyses consists of 6905 observations (61% female, mean age = 21.83, SD age = 3.23). For more information on both the total sample and country samples, see Supplementary Table S1 Additional file 1.

### Measures

#### Face mask use

To assess face mask use, we used the following question to construct our dependent variable: “In the past two months, which of the following measures did you follow and to which extent? Please indicate to what extent you disagree or agree with these statements.” Several statements related to COVID-19 regulations followed, of which one was ‘I used a facemask’. Answers were given on a scale of 1 (Strongly disagree) to 5 (Strongly agree).

#### Risk Perception COVID-19

##### Self-related risk perception COVID-19

Based on the Health Belief Model, we assessed perceived susceptibility and severity [34] by asking: ‘What do you think the likelihood is that in the next two months:’ (1) ‘You get infected with the coronavirus?’ and (2) ‘You must be hospitalized if you are infected with the coronavirus?’. We took an average of the two items. Answers could be given on a scale ranging from 1 (No chance at all) to 7 (Absolutely certain).

##### Other-related risk perception COVID-19

The same two questions but then related to the risk of COVID-19 for family and friends were asked: ‘What do you think the likelihood is that in the next two months: (1) Your family or friends get infected with the coronavirus?’ and ‘(2) Your family or friends must be hospitalized if they are infected with the coronavirus?’. We took an average of the two items. Answers could be given on a scale ranging from 1 (No chance at all) to 7 (Absolutely certain).

#### Experience with COVID-19

##### Direct experience COVID-19

We asked whether participants had been infected with COVID-19, giving the following answer options: ‘Yes, I tested positive’, ‘I think I am/have been infected, but I have not been tested’, and ‘No, I have not been infected or have not been aware of it’. The first two answer options were recoded as ‘1’ and the last answer option as ‘0’ to create a dummy variable indicating direct experience with COVID-19. We chose to combine the two categories as testing capacity was limited and not openly accessible at the time of data collection in most countries.

##### Indirect experience COVID-19

We asked whether friends or family had been infected with the coronavirus, giving the following answer options: ‘Yes, one or more of them tested positive’, ‘Yes, one or more of them think they have been infected but have not been tested’, and ‘No, they have not been infected or have not been aware of it’. The first two answer options were recoded as ‘1’, and the last answer option as ‘0’, to create a dummy variable indicating indirect experience with COVID-19.^1^

#### Attitude towards government

##### Government trust

We asked about general trust in the government of the country: ‘In general, how much trust do you personally have in the [Country] Government on a scale of 1 (no trust at all) to 10 (full trust)?’

##### Perceived clarity communication government

We asked: ‘To what extent do you think the communication from the [Country] Government regarding the measures is clear?’. Answers could be given on a scale from 1 (extremely unclear) to 7 (extremely clear).

#### Policy stringency face mask regulations

##### Stringency face mask regulations

**Fig. 1:**
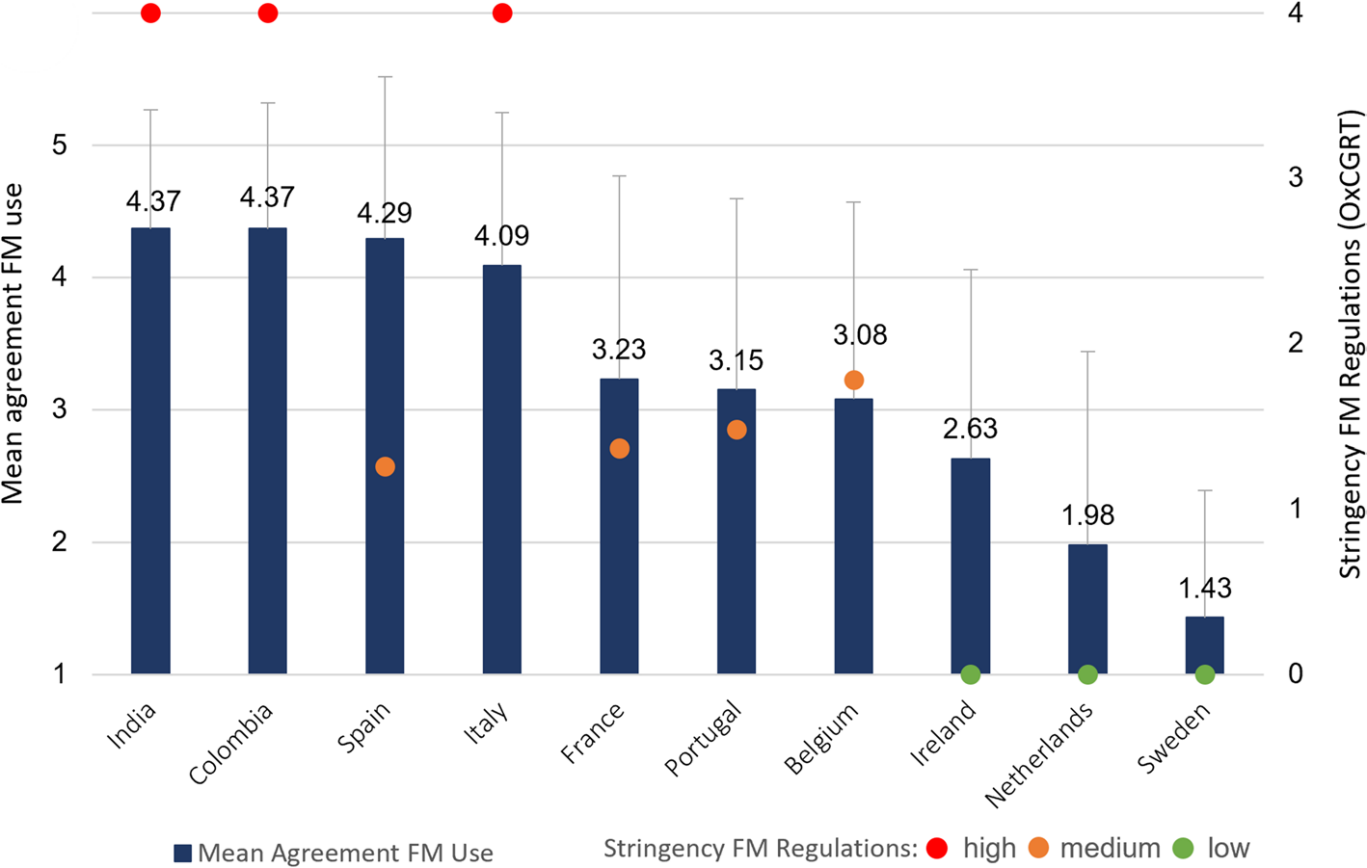
Mean agreement face mask (FM) use ranked from high (5) to low (1) and stringency face mask regulations (0–4; including categorization) across countries

To assess face mask policy stringency, we used data from the Oxford COVID-19 Government Response Tracker (OxCGRT), which consists of systematically collected data on a broad range of COVID-19-related government responses across countries on a day-to-day basis [59]. To assess face mask regulations, we used index H6, which recorded policies on the use of facial coverings outside the home on a daily basis for each country using an ordinal scale from 0 to 4. Policies were scored as follows: 0: no policy; 1: Recommended; 2: Required in some specified shared/public spaces outside the home with other people present, or some situations when social distancing is not possible; 3: Required in all shared/public spaces outside the home with other people present or all situations when social distancing not possible and 4: Required outside the home at all times regardless of location or presence of other people. For each country, we took the index average over the period the survey was online and the subsequent 14 days, as the measures are often communicated before they were initiated.^2^ The stringency score of each country can be found in Fig. 1 and Supplementary Table S2. For the interaction analyses, in which we distinguish between the effects of having no regulation to some regulations and from some regulations to most strict regulations, we categorized the stringency measure. Countries were divided into three groups: low stringency (score ‘0’, Ireland, Netherlands, Sweden), indicating that there was no policy regarding face masks; medium stringency (score 1–3, Belgium, France, Portugal, Spain), indicating that there were intermediate face mask regulations in-between the two “extreme” settings; and high stringency (score ‘4’, Colombia, India, Italy), indicating that a strict policy meaning face masks are required outside the home at all times. This grouping can be found in Fig. 1 (see results).

#### Control variables

We controlled for gender (1: female; 0: male) and age (in years), as both have been related to compliance with COVID-19 protective measures [57, 60]. Moreover, as we are interested in concepts that are strongly linked to the country, such as government trust and country regulations, we controlled for being an international versus domestic student. First, it is likely that government trust and the perceived clarity of government communication differ between international and domestic students because international students may have a different frame of reference, experience language barriers, and may be still very new to the country. Second, international students may still be strongly tied to their home country and therefore potentially exposed to different severities of COVID-19 and different COVID-19-related regulations that apply in the home country. Therefore, we asked students whether they had lived in the country where they attend university for more than 5 years. We infer that those who answered ‘yes’ are domestic students (value 0), while those who answered ‘no’ are international students (value 1).

### Methodology

We treat our dependent variable as a continuous variable – facilitating the interpretation of the coefficients – and perform linear multilevel regressions due to the hierarchical structure of the data (students nested within countries). In addition, multilevel regressions enable an investigation of explained variations at both the individual and country level. The intraclass correlation is .32, which indicates that 32% of the variation in the dependent variable resides at the country level, which is high [61]. Because of the relatively low number of countries, we use restricted maximum likelihood with Kenward-Roger standard errors [62, 63]. Our final sample consists of 6905 observations in ten countries. Analyses were performed using Stata 16.1.

**Fig. 2:**
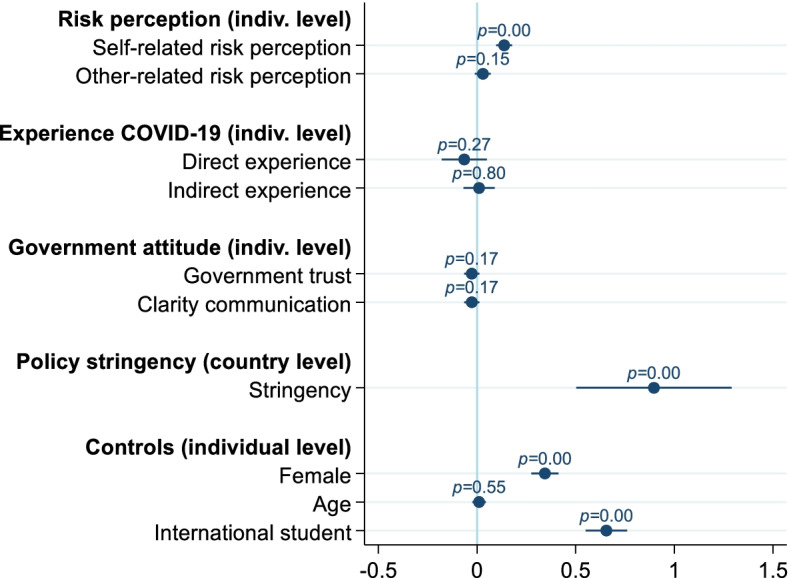
Estimation results of Model 2, Table 3 Values of estimated coefficients are shown, together with their 95% confidence intervals

As a robustness check we replicated Fig. 2 (see results section) with the marginal effects that are retrieved after performing a multilevel ordered logit regression, which takes the ordered nature of the five answer categories into account (but the Kenward-Roger standard errors cannot be calculated). Marginal effects indicate the changes in the probability of answering “strongly agree” (the highest category) for our dependent variable as the result of one-unit increases in the independent and control variables. The marginal effects are shown in Supplementary Fig. S1, and as a percentage of the relative frequency of “strongly agree” in the sample (i.e., 0.24).

#### Descriptive statistics

Table 1 provides the descriptive statistics of the variables. The regression analyses contain standardized variables only (the 1/0 variables are not standardized).

**Table 1:** Means and standard deviations individual-level variables

	Mean	SD	Min.	Max.
Face mask use (1 – Strongly Disagree to 5 – Strongly agree)	3.00	1.57	1	5
Self-related risk perception COVID-19 (1 – No chance at all to 7 – Absolute certain)	3.36	1.00	1	7
Other-related risk perception COVID-19 (1 – No chance at all to 7 – Absolute certain)	4.31	1.02	1	7
Direct experience COVID-19 (1 – Yes; 0 – No)	0.10	0.30	0	1
Indirect experience COVID-19 (1 – Yes; 0 – No)	0.29	0.45	0	1
Government trust (1 – Low to 10 – High)	5.96	2.22	1	10
Perceived clarity government communication (1 – Extremely unclear to 7 – Extremely clear)	4.54	1.55	1	7
Stringency face mask regulations (0 – No policy to 4 – Required everywhere at all times)	1.79	1.66	0	4
Gender (1 – Female; 0 – Male)	0.61	0.49	0	1
Age (in years)	21.83	3.23	17	35
International student (1 – Yes; 0 – No)	0.12	0.33	0	1

SD=standard deviation. Table based on 6905 observations. Statistics based on the unstandardized variables. Mean and SD of the stringency variable at the country level based on 10 countries

Table 2 presents the correlation matrix between the individual-level variables. Correlations are generally low (below ±.10), apart from a few exceptions. We also calculated the variance inflation factors, and they did not exceed 1.5 for any variable (not reported).

**Table 2:**
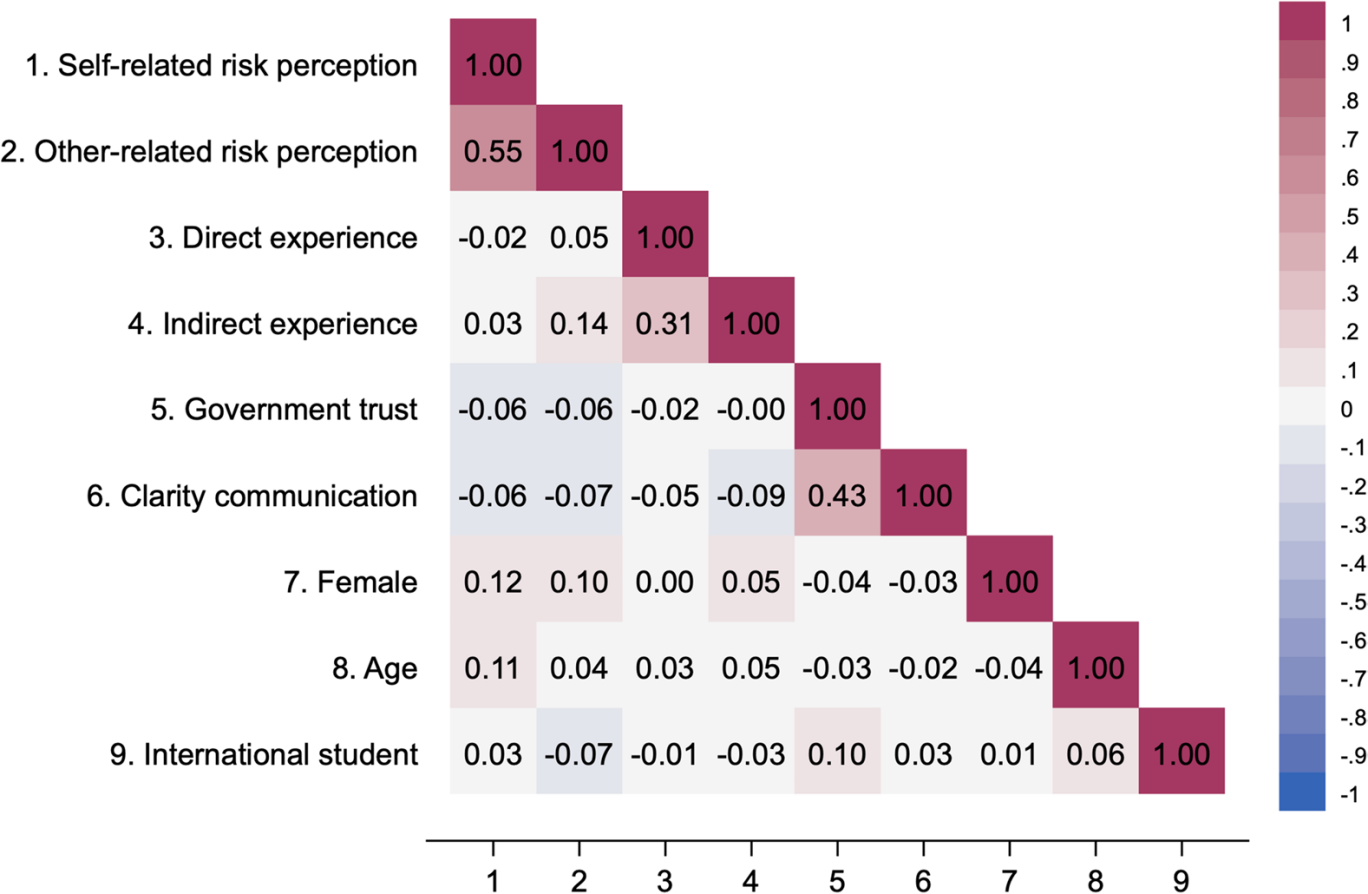
Correlation matrix (individual-level variables)

Numbers are based on 6905 observations. Pearson correlations are displayed

## Results

### Face mask use and regulations across countries

Figure 1 presents the means of our face mask-wearing measure (the dependent variable) across countries. A higher value indicates higher agreement and higher usage of face masks. Mean values are represented by the blue vertical bars in Fig. 1 and presented above the bars. The stringency of face mask regulations for each country based on the OxCGRT is indicated by the circles. By categorizing the countries, we can more easily draw conclusions on the effects of different types of regulations. The categorization is indicated by the different colors of the circles in Fig. 1 (green: low – no regulations/recommendations, orange: medium – intermediate regulations, red: high – strict regulations). Exact values and standard deviations are presented in Supplementary Table S2.

We note large differences in face mask usage across countries in our data, with average agreement per country ranging from 1.43 to 4.37. Colombian and Indian students indicated the highest agreement with face mask use, whereas agreement was lowest among Dutch and Swedish students. French students showed the highest variation in agreement with using face masks. Finally, Fig. 1 shows that countries without regulations (the Netherlands, Ireland, and Sweden) had the lowest average agreement with face mask use.

### Individual-level variables and face mask use

We performed linear multilevel regressions with face mask use as the dependent variable (Hox et al., 2017).

Model 1, presented in Table 3, only includes the country-level random intercept. Model 2 of Table 3 includes all control variables and independent variables. Figure 2 graphically summarizes the results of Model 2.

**Table 3:** Linear multi-level regressions with face mask use as the dependent variable

	Model 1			Model 2			Model 3		
	Coeff.	SE	*p*-value	Coeff.	SE	*p*-value	Coeff.	SE	*p*-value
Intercept	**3.26**	**0.31**	**< 0.001**	**2.98**	**0.19**	**< 0.001**	**1.68**	**0.28**	**< 0.001**
*Risk perception COVID-19 (individual level)*									
Self-related				**0.14**	**0.02**	**< 0.001**	**0.14**	**0.02**	**< 0.001**
Other-related				0.03	0.02	0.15	0.03	0.02	0.15
*Experience COVID-19 (individual level)*									
Direct experience				−0.06	0.06	0.27	−0.06	0.06	0.27
Indirect experience				0.01	0.04	0.80	0.01	0.04	0.80
*Government attitude (individual level)*									
Government trust				−0.03	0.02	0.17	− 0.03	0.02	0.17
Perceived clarity communication				−0.03	0.02	0.17	−0.03	0.03	0.18
*Policy stringency (country level)*									
Stringency facemask regulations				**0.90**	**0.20**	**0.002**			
Stringency: medium (vs. low)							**1.48**	**0.37**	**0.005**
Stringency: high (vs. low)							**2.35**	**0.40**	**< 0.001**
*Controls (individual level)*									
Female				**0.34**	**0.04**	**< 0.001**	**0.34**	**0.04**	**< 0.001**
Age				0.01	0.02	0.55	0.01	0.01	0.54
International student				**0.66**	**0.05**	**< 0.001**	**0.66**	**0.05**	**< 0.001**
Variance individual level	2.02	0.03		1.92	0.03		1.92	0.03	
Variance country level	0.95	0.43		0.35	0.18		0.23	0.13	
Pseudo *R*^2^ individual level				0.05			0.05		
Pseudo *R*^2^ country level				0.63			0.76		
Deviance	24,496			24,166			24,160		
AIC / BIC	24,502	24,523		24,192	24,281		24,188	24,284	
Number of individuals	6905			6905			6905		
Number of countries	10			10			10		

SE=Kenward-Roger standard error. Restricted maximum likelihood is used. Estimates in bold represent *p*-values< 0.05. Each model includes a country-level random intercept

For Model 2, we reported the change in the unexplained variance at the individual and country levels relative to Model 1 (pseudo *R*^2^). The individual-level variables explained approximately 5% of the variation at the individual level; the country variable explained 63% of the variation at the country level. We also reported the deviance statistic for each model, where a lower value indicates better model fit. Regarding the control variables, we noted that women were significantly more likely to report wearing a face mask than men and that international students (i.e., students studying not in their country of origin) were significantly more likely to report wearing a face mask than domestic students.

#### Risk perception COVID-19

We noted that self-related risk perception of COVID-19 was positively and significantly associated with face mask use (*p* < .001). A standard deviation increase in this standardized measure is expected to improve agreement with face mask use by 0.14 points. Other-related risk perception of COVID-19 (perceived risk of COVID-19 for family and friends) is not significantly related to face mask use (*p* = .15). The associated coefficient is approximately four times smaller than the coefficient of self-related risk perception (a Wald test for the equality of coefficients results in *p* = .003).

#### Experience with COVID-19

We do not find a significant association between direct (*p* = .27) or indirect experience (*p* = .80) with COVID-19 and agreement to use a face mask.

#### Attitude towards government

The individual-level governmental variables did not significantly explain face mask use (*p* = .17 for both variables).

#### Policy stringency

Including the stringency variable at the country level as a continuous variable (Model 2, Table 3) showed a strong positive association between stringency of face mask regulations and agreement with face mask use. A one-standard-deviation increase in this standardized measure is expected to increase agreement with face mask use by 0.90 points. Model 3, Table 3 includes the categorized stringency measure (low: no regulations, medium: intermediate regulations, high: strict regulations), showing that both higher and medium stringency of regulations compared to the reference category (low stringency) was significantly positively associated with agreement with face mask use (low; *p* = .005 for medium, and *p* < .001 for high). A Wald test on the difference between the coefficients of the medium and high stringency dummy variables resulted in *p* = .019 (not reported in Table 3). Hence, students were not only more likely to agree with face mask use in countries with some measures implemented (relative to none) but were also more likely to wear face masks in countries with strict regimes than in countries with some intermediate regime. The effect sizes of the regimes in terms of the implied point differences are substantial, that is, they reflect increases of 49% (intermediate regulations) and 78% (strict regulations) relative to the mean of the dependent variable (which is 3.00).

### Differences across face mask policy stringency levels

We next focused on how the impact of the individual-level variables differed across countries with different stringency of regulations based on the policy stringency variable. We consecutively added interaction terms between each individual-level variable and the categorical country-level policy stringency variable. Next to our variables of interest, we also added interaction terms between the control variables and the policy stringency variable. A random slope for the specific individual-level variable was added, together with a covariance term between the random intercept and random slope [64].

**Fig. 3:**
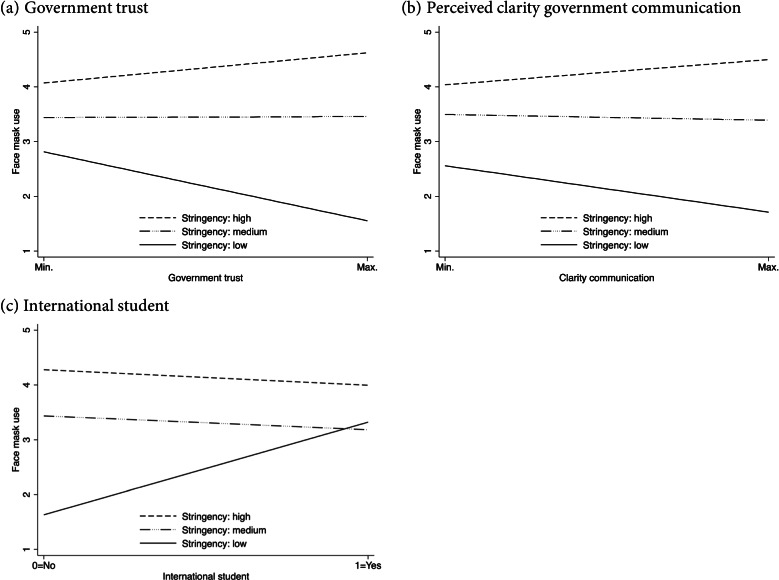
Interaction Plots Based on Table 4

For three variables, we found significant coefficients of the interaction terms: government trust, perceived clarity of government communication, and the international student variable. For the other individual-level variables no statistically significant interaction coefficients were found. Table 4 contains these three models and shows the statistically significant interaction coefficients: Model 1 includes interaction terms between government trust and stringency, Model 2 includes interaction terms between perceived clarity of communication and stringency, and Model 3 includes interaction terms between the international student variable and stringency. Supplementary Table S3 in Additional File 1 shows the regression results for the variables not included in Table 4 and Fig. 3 Supplementary Fig. S3 displays the interaction plots based on Supplementary Table S3.

**Table 4:** Linear multi-level regressions with face mask use as the dependent variable (including interactions)

	Model 1 Interactions with Government trust			Model 2 Interactions with Perceived clarity communication			Model 3 Interactions with International student		
	Coeff.	SE	*p*-value	Coeff.	SE	*p*-value	Coeff.	SE	*p*-value
Intercept	**1.83**	**0.29**	**< 0.001**	**1.77**	**0.29**	**< 0.001**	**1.43**	**0.28**	**0.001**
*Risk perception COVID-19 (individual level)*									
Self-related	**0.14**	**0.02**	**< 0.001**	**0.14**	**0.02**	**< 0.001**	**0.12**	**0.02**	**< 0.001**
Other-related	0.03	0.02	0.16	0.03	0.02	0.14	0.03	0.02	0.19
*Experience COVID-19 (individual level)*									
Direct experience	− 0.08	0.06	0.20	−0.07	0.06	0.24	−0.02	0.06	0.75
Indirect experience	0.02	0.04	0.65	0.01	0.04	0.76	0.03	0.04	0.50
*Government attitude (individual level)*									
Government trust	**−0.31**	**0.05**	**< 0.001**	− 0.03	0.02	0.18	0.005	0.02	0.79
Perceived clarity communication	−0.02	0.02	0.23	**−0.22**	**0.06**	**0.01**	−0.01	0.02	0.77
*Policy stringency (country level)*									
Stringency: medium (vs. low)	**1.33**	**0.38**	**0.01**	**1.37**	**0.38**	**0.01**	**1.81**	**0.36**	**0.002**
Stringency: high (vs. low)	**2.25**	**0.41**	**< 0.001**	**2.25**	**0.41**	**< 0.001**	**2.65**	**0.39**	**< 0.001**
*Interactions*									
Government trust × Stringency: medium (vs. low)	**0.32**	**0.07**	**0.01**						
Government trust × Stringency: high (vs. low)	**0.45**	**0.08**	**< 0.001**						
Perc. clarity communication × Stringency: medium (vs. low)				0.19	0.07	0.06			
Perc. clarity communication × Stringency: high (vs. low)				**0.34**	**0.08**	**0.001**			
International student × Stringency: medium (vs. low)							**−1.95**	**0.29**	**0.005**
International × Stringency: high (vs. low)							**−1.98**	**0.45**	**< 0.001**
*Controls (individual level)*									
Female	**0.35**	**0.04**	**< 0.001**	**0.35**	**0.04**	**< 0.001**	**0.33**	**0.03**	**< 0.001**
Age	0.01	0.02	0.53	0.01	0.02	0.59	0.03	0.02	0.05
International student	**0.61**	**0.05**	**< 0.001**	**0.62**	**0.05**	**< 0.001**	**1.69**	**0.22**	**0.003**
Variance individual level	1.90	0.03		1.91	0.03		1.83	0.03	
Variance country level	0.24	0.13		0.24	0.13		0.22	0.12	
Variance random slope	0.001	0.003		0.002	0.003		0.07	0.08	
Covariance	−0.002	0.02		−0.02	0.02		−0.11	0.12	
Pseudo *R*^2^ individual level	0.06			0.06			0.09		
Pseudo *R*^2^ country level	0.75			0.75			0.77		
Deviance	24,117			24,141			23,857		
AIC / BIC	24,154	24,277		24,177	24,300		23,893	24,016	
Number of individuals	6905			6905			6905		
Number of countries	10			10			10		

SE*=*Kenward-Roger standard error. Restricted maximum likelihood is used. Estimates in bold represent *p*-values< 0.05. Each model includes a random intercept term, a random slope term (for government trust in Model 1, perceived clarity in Model 2, and international student in Model 3), and a covariance term between intercept and slope

For ease of interpretation, Fig. 3 shows the interaction plots based on Models 1, 2, and 3 of Table 4. Figure 3a (government trust) and 3b (perceived clarity of communication) show that higher values for government trust and perceived clarity communication are associated with higher agreement levels to wear face masks in relatively strict countries regarding face mask use. As expected, trust in government and perceived clarity of governmental communication significantly increased face mask use in the most stringent countries (Wald tests: *p* = .014 for trust and *p* < .001 for communication) and significantly decreased face mask use in the least stringent countries (*p* < .001 for trust (Model 1, Table 4) and *p* = .01 for communication (Model 2, Table 4)). Furthermore, Fig. 3c shows that international students were significantly more likely to wear face masks than domestic students in countries without face mask recommendations or requirements. Specifically, this relationship was not significant in countries with medium (*p* = .112) and high stringency (*p* = .455). T-tests revealed that international students were more likely to trust the national government of the country where they study – *M*(internationals) = 6.59; *M*(domestic) = 5.88; *p* < .001 – and were more positive about the government’s communication: *M*(internationals) = 4.66; *M*(domestic) = 4.52; *p* = .02. Because of these differences between international and domestic students we replicated our main results for the sample excluding international students (6065 observations). See Supplementary Fig. S2.

Supplementary Table S4 provides a robustness test of the interaction effects by performing an OLS regression with country dummy variables included (and with cluster-robust standard errors). The results for the interaction terms were qualitatively similar to those in Table 4; the same holds for the other individual-level variables.

## Discussion

In 2021, COVID-19 vaccines and treatments have become widely available in rich countries. However, vaccination rates have remained low in some countries, and even in countries with high vaccination rates, new peaks of infection have emerged due to novel and more infectious variants. Moreover, poorer countries usually cannot afford large-scale vaccination. Consequently, many countries still need to rely on face masks and distancing (with lockdowns in extremis) as the main medical precautions. Since face mask usage is economically cheap and less disruptive compared to other regulations, such as social distancing and lockdowns, knowledge about the motives for using them is essential. Moreover, as countries may lower the stringency of their measures, it is interesting to know whether this affects the relationship between mask-use and individual level variables that were found to be important in earlier literature.

Our analyses of almost 7000 students in ten countries during the early phase of the 2020 COVID-19 pandemic show that the stringency of regulations in a country is most strongly related to face mask use, with stricter rules associated with stricter face mask use. In distinguishing between the relative stringency of face mask regulations, we show that not only does imposing any regulations relative to no regulations relate to a higher agreement with face mask use but installing strict regulations relative to intermediate regulations also increases agreement. We also find that self-related risk perception of COVID-19 positively relates to agreement with face mask use, while other-related risk perception of COVID-19 did not relate to face mask use. This is in contrast with studies showing that social risk perception affects compliance [36, 37] and studies that show that inducing empathy for vulnerable people and stressing prosocial consequences of mask-wearing is related to a higher motivation to wear a mask [36, 65]. Moreover, against expectation, we do not find a relationship between attitude towards government and (in)direct experience with COVID-19 infection and agreement with face mask use.

Analyzing the interaction between policy stringency and our individual level factors, we find an interaction effect between policy stringency and attitude towards government. A more positive attitude towards government increases face mask use in stringent countries and decreases face mask use in countries without recommendations or requirements. The finding of an interaction between government trust and policy stringency is in line with the findings of others that studied compliance with other COVID-19 related preventive measures [10, 52]. We are the first to show that the same relationship is present between stringency and perceived clarity of government communication, meaning that the link between stringency and face mask use becomes stronger when communication is clearer. Our distinction in low (no regulations), medium and high stringency allows us to draw the conclusion that in a situation without any regulations trust and perceived clarity of communication negatively associate with mask use. In countries without face mask regulations or recommendations, governments did not explicitly advise against the use but did openly question the scientific basis for their effectiveness which may have conveyed a negative attitude towards masks. Hence, a more positive government perception relates to lower face mask use in these countries and to higher use in countries with such regulations. As stated, both obedience to authority and conformity through social pressure may underlie the importance of regulations [14, 66]. People are in general obedient when it comes to people of power [14]. At the same time, behavior is contagious. When governments impose face mask regulations, this enforces a social norm that subsequently stimulates the advocated behavior because people want to conform to the group standard [66, 67].

We did not find an interaction between policy stringency and risk perception or experience with COVID-19. We expected that individual differences in perceptions and experiences would play a smaller role in countries with strict regulations, as these are ‘strong’ situations in which the range of acceptable behavior is limited. Nevertheless, it seems that in our sample experience with COVID-19 is not associated with mask usage across all regulation regimes, while the positive association between self-related risk perception of COVID-19 and mask use is present across all policy stringency contexts.

A limitation of this research is that we use self-report data of face mask use. Previous research shows that self-report measures vary in their correspondence to actual behaviour [68, 69]. While responses were provided anonymously in our survey, it is conceivable that they are subject to social desirability bias. However, recall bias is likely to be low, because the saliency of the pandemic and novelty of face mask use as a behaviour may have made it easier to recall it. Moreover, Petherick et al. [70] found that survey data on compliance with physical distancing during the pandemic was related to objective mobile-phone mobility data. If a similar situation occurs in the future, collecting more objective measures of face mask use would be worthwhile. Since relevant data could only be collected during a limited time frame, this was outside the options and scope of our research project.

Besides this, the study is limited in that we studied a set of countries that do not cover a random and representative sample of the global population. We focus on factors associated with face mask use among students, a group that represents a specific subsample of the total population with on the one hand below-average incentives for protective behaviors compared to older generations, and on the other hand above-average levels of rule abidance compared to those with an average education [71]. The results should therefore not be generalized to other populations. Since the data were collected at the very beginning phase of the COVID-19 pandemic, further research is needed to study the effects of regulations changing over time and whether perceptions of risk and perceived benefits of face mask use shift over time during a long-lasting pandemic.

As face mask use is only an efficient method to lower the spread of COVID-19 if there is widespread adoption [72], governments should put country-wide regulations in place if they decide to involve face masks to halt the pandemic. Our study shows that the stringency of regulations is most strongly associated with face mask use among students. The strength of this relationship can be further increased by clear government communication and enhancing government trust. From our study, it appears that self-related risk perception of COVID-19 is also important for face mask use, while other-related risk perception, direct and indirect experience with COVID-19 are not associated with mask use at all.

## Supplementary Information

**Additional file 1.** Supplementary Tables and Figures.

## Data Availability

The datasets generated and/or analysed during the current study are available in the Erasmus University Rotterdam (EUR) Data Repository, 10.25397/eur.19923062.

## Footnotes

1 Additional analyses disaggregating the indirect experience with COVID-19 variable into confirmed COVID-19 and “I think I had it” leads to the same results and conclusions. For the direct experience with COVID-19 variable, the number of observations in the confirmed COVID-19 category was too small (*n* = 17) to conduct reliable sensitivity analyses.

2 We conducted robustness analyses using alternative calculations for the face mask policy stringency variable (taking the index average of the same period but then a) including the 14 days before the survey period; and b) including the two months before the survey period). These analyses led to similar results and conclusions. The categorization of the countries using the alternative stringency variables remains identical.
