# Supplementary material for: Face mask use during the COVID-19 pandemic: how risk perception, experience with COVID-19, and attitude towards government interact with country-wide policy stringency
Source: BMC Public Health. 2022 Aug 26;22:1622. doi: 10.1186/s12889-022-13632-9 (PMC9412789; doi:10.1186/s12889-022-13632-9)
Supplement: Supplementary file 1 — Additional file 1. Supplementary Tables and Figures. [file 12889_2022_13632_MOESM1_ESM.docx]

Supplementary Material

Supplementary Table S1. Descriptive statistics per country.

|  |  | NL | BE | PRT | ESP | IND | FR | SWE | IT | IRE | COL | Total |
| --- | --- | --- | --- | --- | --- | --- | --- | --- | --- | --- | --- | --- |
| Sample size | *N* | 1066 | 3363 | 1178 | 146 | 183 | 200 | 234 | 188 | 90 | 257 | 6905 |
| Female | *%* | 57.4% | 68.1% | 47.6% | 66.4% | 45.4% | 73.0% | 56.4% | 53.7% | 65.6% | 63.0% | 61.4% |
| Age (in years) | *M*  (*SD*) | 20.63 (1.98) | 21.95 (3.29) | 21.77 (3.23) | 22.51 (3.17) | 21.37 (2.98) | 20.55 (2.18) | 24.82 (3.86) | 22.48 (2.28) | 22.54 (3.54) | 23.04 (4.47) | 21.83 (3.23) |
| International student | *%* | 30.4% | 10.1% | 7.8% | 2.7% | 2.2% | 7.5% | 9.0% | 5.9% | 32.2% | 0.4% | 12.2% |
| Self-related risk perception COVID-19 (1–7) | *M*  (*SD*) | 3.23 (0.89) | 3.40 (1.01) | 3.18 (0.93) | 3.07 (0.99) | 4.04 (1.19) | 3.76 (1.05) | 3.51 (0.91) | 3.32 (0.95) | 3.17 (1.01) | 3.48 (1.18) | 3.36 (1.00) |
| Other-related risk perception COVID-19 (1–7) | *M*  (*SD*) | 4.08 (0.94) | 4.48 (0.99) | 4.10 (0.96) | 3.92 (1.16) | 4.40 (1.25) | 4.56 (1.10) | 4.34 (0.90) | 4.02 (0.98) | 4.27 (1.05) | 4.17 (1.21) | 4.31 (1.02) |
| Direct experience COVID-19 | *%* | 12.9% | 12.5% | 3.7% | 4.1% | 0.5% | 8.5% | 19.2% | 6.4% | 10.0% | 2.7% | 10.2% |
| Indirect experience COVID-19 | *%* | 31.4% | 38.0% | 10.1% | 24.7% | 0.5% | 29.0% | 42.3% | 18.6% | 32.2% | 2.7% | 28.9% |
| Government trust (1–10) | *M*  (*SD*) | 7.29 (1.63) | 5.58 (2.11) | 6.42 (2.00) | 4.18 (2.55) | 6.19 (2.50) | 5.58 (2.07) | 7.03 (2.29) | 5.55 (2.22) | 6.94 (1.86) | 3.61 (2.16) | 5.96 (2.22) |
| Perceived clarity government communication (1–7) | *M*  (*SD*) | 5.13 (1.31) | 4.08 (1.49) | 5.32 (1.25) | 3.53 (1.80) | 4.90 (1.75) | 4.16 (1.63) | 4.86 (1.60) | 4.14 (1.50) | 5.66 (1.38) | 4.78 (1.45) | 4.54 (1.55) |

M=Mean; SD=standard deviation.

Supplementary Table S2. Mean and standard deviations (SD) of agreement with face mask (FM) use (1-5) and stringency face mask regulations (0-4) across countries.

| Country | Mean agreement FM use | SD agreement FM use | Observations | Stringency  FM regulations (0-4) | Categorization stringency FM regulations |
| --- | --- | --- | --- | --- | --- |
| India | 4.37 | 0.90 | 183 | 4 | high |
| Colombia | 4.37 | 0.95 | 257 | 4 | high |
| Spain | 4.29 | 1.23 | 146 | 1.26 | medium |
| Italy | 4.09 | 1.16 | 188 | 4 | high |
| France | 3.23 | 1.54 | 200 | 1.37 | medium |
| Portugal | 3.15 | 1.45 | 1,178 | 1.48 | medium |
| Belgium | 3.08 | 1.49 | 3,363 | 1.78 | medium |
| Ireland | 2.63 | 1.43 | 90 | 0 | low |
| Netherlands | 1.98 | 1.46 | 1,066 | 0 | low |
| Sweden | 1.43 | 0.96 | 234 | 0 | low |
| Total | 3.00 | 1.57 | 6,905 |  |  |

**Supplementary Table S3. Linear multi-level regressions with face mask use as the dependent variable.**

|  | | Interactions with Female | | | Interactions with Age | | | Interactions with Self-related risk perception COVID-19 | | |
| --- | --- | --- | --- | --- | --- | --- | --- | --- | --- | --- |
|  | | Coeff. | SE | *p*-value | Coeff. | SE | *p*-value | Coeff. | SE | *p*-value |
| Intercept | | **1.73** | **0.28** | **<0.001** | **1.69** | **0.28** | **<0.001** | **1.68** | **0.28** | **<0.001** |
| Risk perception COVID-19 (individual level) | | | | |  |  |  |  |  |  |
| Self-related | | **0.14** | **0.02** | **<0.001** | **0.14** | **0.02** | **<0.001** | 0.11 | 0.05 | 0.09 |
| Other-related | | 0.03 | 0.02 | 0.15 | 0.03 | 0.02 | 0.15 | 0.03 | 0.02 | 0.1 |
| Experience COVID-19 (individual level) | | | |  |  |  |  |  |  |  |
| Direct experience | | -0.06 | 0.06 | 0.27 | -0.07 | 0.06 | 0.26 | -0.06 | 0.06 | 0.28 |
| Indirect experience | | 0.01 | 0.04 | 0.83 | 0.01 | 0.04 | 0.78 | 0.01 | 0.04 | 0.85 |
| Government attitude (individual level) | | | |  |  |  |  |  |  |  |
| Government trust | | -0.03 | 0.02 | 0.16 | -0.03 | 0.02 | 0.18 | -0.03 | 0.02 | 0.16 |
| Perceived clarity communication | | -0.03 | 0.02 | 0.19 | -0.03 | 0.02 | 0.17 | -0.03 | 0.02 | 0.19 |
| Policy stringency (country level) | | | |  |  |  |  |  |  |  |
| Stringency: medium (vs. low) | | **1.39** | **0.37** | **0.01** | **1.47** | **0.37** | **0.01** | **1.47** | **0.37** | **0.005** |
| Stringency: high (vs. low) | | **2.35** | **0.4** | **<0.001** | **2.34** | **0.4** | **<0.001** | **2.38** | **0.4** | **<0.001** |
| Interactions | |  |  |  |  |  |  |  |  |  |
| Female × Stringency: medium | | 0.14 | 0.12 | 0.34 |  |  |  |  |  |  |
| Female × Stringency: high | | -0.01 | 0.14 | 0.97 |  |  |  |  |  |  |
| Age × Stringency: medium | |  |  |  | 0.05 | 0.07 | 0.44 |  |  |  |
| Age × Stringency: high | |  |  |  | 0.09 | 0.08 | 0.27 |  |  |  |
| Self-related risk × Stringency: medium |  | | |  |  |  |  | 0.03 | 0.07 | 0.66 |
| Self-related risk × Stringency: high |  | | |  |  |  |  | -0.08 | 0.08 | 0.29 |
| Controls (individual level) | | |  |  |  |  |  |  |  |  |
| Female | | **0.25** | **0.09** | **0.04** | **0.34** | **0.03** | **<0.001** | **0.34** | **0.04** | **<0.001** |
| Age | | 0.01 | 0.02 | 0.52 | -0.05 | 0.05 | 0.31 | 0.01 | 0.02 | 0.6 |
| International student | | **0.66** | **0.05** | **<0.001** | **0.66** | **0.05** | **<0.001** | **0.66** | **0.05** | **<0.001** |
| Variance individual level | | 1.92 | 0.03 |  | 1.92 | 0.03 |  | 1.91 | 0.03 |  |
| Variance country level | | 0.22 | 0.13 |  | 0.23 | 0.13 |  | 0.23 | 0.13 |  |
| Variance random slope | | 0 | 0 |  | 0.001 | 0.002 |  | 0.001 | 0.003 |  |
| Covariance | | 0.004 | 0.004 |  | -0.01 | 0.02 |  | -0.015 | 0.02 |  |
| Pseudo *R*^2^ individual level | | 0.05 |  |  | 0.05 |  |  | 0.05 |  |  |
| Pseudo *R*^2^ country level | | 0.79 |  |  | 0.78 |  |  | 0.79 |  |  |
| Deviance | | 24163 |  |  | 24166 |  |  | 24161 |  |  |
| AIC / BIC | | 24199 | 24322 |  | 24202 | 24325 |  | 24197 | 24320 |  |
| Number of individuals | | 6,905 |  |  | 6,905 |  |  | 6,905 |  |  |
| Number of countries | | 10 |  |  | 10 |  |  | 10 |  |  |

SE= Kenward-Roger standard error. Restricted maximum likelihood is used. Estimates in bold represent p-values<0.05.

Supplementary Table S3 (continued). Linear multi-level regressions with face mask use as the dependent variable.

|  | Interactions with Direct experience COVID-19 | | | Interactions with Other-related risk perception COVID-19 | | | Interactions with Indirect experience COVID-19 | | |
| --- | --- | --- | --- | --- | --- | --- | --- | --- | --- |
|  | Coeff. | SE | *p*-value | Coeff. | SE | *p*-value | Coeff. | SE | *p*-value |
| Intercept | **1.7** | **0.29** | **<0.001** | **1.68** | **0.28** | **<0.001** | **1.77** | **0.29** | **<0.001** |
| Risk perception COVID-19 (individual level) | | | |  |  |  |  |  |  |
| Self-related | **0.14** | **0.02** | **<0.001** | **0.14** | **0.02** | **<0.001** | **0.14** | **0.02** | **<.001** |
| Other-related | 0.03 | 0.02 | 0.14 | -0.06 | 0.07 | 0.40 | 0.03 | 0.02 | 0.13 |
| Experience COVID-19 (individual level) | | |  |  |  |  |  |  |  |
| Direct experience | -0.22 | 0.21 | 0.37 | -0.06 | 0.06 | 0.28 | -0.07 | 0.06 | 0.25 |
| Indirect experience | 0.01 | 0.04 | 0.85 | 0.004 | 0.04 | 0.93 | -0.24 | 0.11 | 0.15 |
| Government attitude (individual level) | | |  |  |  |  |  |  |  |
| Government trust | -0.03 | 0.02 | 0.19 | -0.03 | 0.02 | 0.16 | -0.03 | 0.02 | 0.20 |
| Perceived clarity communication | -0.03 | 0.02 | 0.15 | -0.03 | 0.02 | 0.19 | -0.03 | 0.02 | 0.18 |
| Policy stringency (country level) | | |  |  |  |  |  |  |  |
| Stringency: medium (vs. low) | **1.46** | **0.38** | **0.01** | **1.47** | **0.37** | **0.005** | **1.38** | **0.38** | **0.01** |
| Stringency: high (vs. low) | **2.35** | **0.41** | **<0.001** | **2.34** | **0.4** | **<0.001** | **2.29** | **0.41** | **<0.001** |
| Interactions |  |  |  |  |  |  |  |  |  |
| Direct experience × Stringency: medium | 0.06 | 0.28 | 0.86 |  |  |  |  |  |  |
| Direct experience × Stringency: high | -0.54 | 0.41 | 0.21 |  |  |  |  |  |  |
| Other-related risk × Stringency: medium | | |  | 0.10 | 0.08 | 0.28 |  |  |  |
| Other-related risk × Stringency: high | | |  | -0.03 | 0.09 | 0.77 |  |  |  |
| Indirect experience × Stringency: medium | | |  |  |  |  | 0.30 | 0.17 | 0.33 |
| Indirect experience × Stringency: high | | |  |  |  |  | -0.17 | 0.26 | 0.52 |
| Controls (individual level) | |  |  |  |  |  |  |  |  |
| Female | **0.34** | **0.04** | **<0.001** | **0.34** | **0.04** | **<0.001** | **0.34** | **0.04** | **<0.001** |
| Age | 0.01 | 0.02 | 0.54 | 0.01 | 0.02 | 0.55 | 0.01 | 0.02 | 0.54 |
| International student | **0.65** | **0.05** | **<0.001** | **0.67** | **0.05** | **<0.001** | **0.65** | **0.05** | **<0.001** |
| Variance individual level | 1.91 | 0.03 |  | 1.91 | 0.03 |  | 1.91 | 0.03 |  |
| Variance country level | 0.23 | 0.13 |  | 0.23 | 0.13 |  | 0.24 | 0.14 |  |
| Variance random slope | 0.05 | 0.05 |  | 0.005 | 0.004 |  | 0.005 | 0.01 |  |
| Covariance | -0.06 | 0.1 |  | -0.02 | 0.02 |  | -0.03 | 0.05 |  |
| Pseudo *R*^2^ individual level | 0.05 |  |  | 0.05 |  |  | 0.05 |  |  |
| Pseudo *R*^2^ country level | 0.78 |  |  | 0.79 |  |  | 0.77 |  |  |
| Deviance | 24151 |  |  | 24150 |  |  | 24147 |  |  |
| AIC / BIC | 24187 | 24310 |  | 24186 | 24309 |  | 24183 | 24306 |  |
| Number of individuals | 6,905 |  |  | 6,905 |  |  | 6,905 |  |  |
| Number of countries | 10 |  |  | 10 |  |  | 10 |  |  |

SE= Kenward-Roger standard error. Restricted maximum likelihood is used. Estimates in bold represent p-values<0.05.

Supplementary Table S4. OLS Regressions with face mask use as the dependent variable (Including significant interactions).

|  | Model 1  Interactions with Government trust | | | Model 2  Interactions with Perceived clarity communication | | | Model 3  Interactions with International student | | |
| --- | --- | --- | --- | --- | --- | --- | --- | --- | --- |
|  | Coeff. | SE | *p*-value | Coeff. | SE | *p*-value | Coeff. | SE | *p*-value |
| Intercept | **2.76** | **0.04** | **<0.001** | **2.73** | **0.04** | **<0.001** | **2.70** | **0.04** | **<0.001** |
| Risk perception COVID-19 (individual level) |  |  |  |  |  |  |  |  |  |
| Self-related | **0.14** | **0.02** | **<0.001** | **0.14** | **0.02** | **<0.001** | **0.12** | **0.02** | **<0.001** |
| Other-related | 0.03 | 0.03 | 0.41 | 0.03 | 0.03 | 0.39 | 0.03 | 0.03 | 0.42 |
| Experience COVID-19 (individual level) |  |  |  |  |  |  |  |  |  |
| Direct experience | -0.07 | 0.10 | 0.46 | -0.07 | 0.09 | 0.49 | -0.02 | 0.07 | 0.82 |
| Indirect experience | 0.02 | 0.05 | 0.50 | 0.01 | 0.05 | 0.80 | 0.02 | 0.04 | 0.58 |
| Government attitude (individual level) |  |  |  |  |  |  |  |  |  |
| Government trust | **-0.32** | **0.05** | **<0.001** | -0.03 | 0.05 | 0.59 | 0.005 | 0.03 | 0.88 |
| Perceived clarity communication | -0.02 | 0.02 | 0.22 | **-0.21** | **0.06** | **0.01** | -0.005 | 0.01 | 0.68 |
| Controls (individual level) |  |  |  |  |  |  |  |  |  |
| Female | **0.35** | **0.03** | **<0.001** | **0.35** | **0.03** | **<0.001** | **0.33** | **0.04** | **<0.001** |
| Age | 0.01 | 0.03 | 0.70 | 0.01 | 0.03 | 0.74 | **0.03** | **0.01** | **0.04** |
| International student | 0.61 | 0.52 | 0.27 | 0.62 | 0.53 | 0.27 | **1.82** | **0.10** | **<0.001** |
| Interactions |  |  |  |  |  |  |  |  |  |
| Government trust × Stringency: medium  (vs. low) | **0.33** | **0.06** | **0.001** |  |  |  |  |  |  |
| Government trust × Stringency: high  (vs. low) | **0.46** | **0.07** | **<0.001** |  |  |  |  |  |  |
| Clarity communication × Stringency: medium (vs. low) |  |  |  | **0.21** | **0.06** | **0.01** |  |  |  |
| Clarity communication × Stringency: high (vs. low) |  |  |  | **0.33** | **0.05** | **<0.001** |  |  |  |
| International student × Stringency: medium (vs. low) |  |  |  |  |  |  | **-1.89** | **0.10** | **<0.001** |
| International student × Stringency: high  (vs. low) |  |  |  |  |  |  | **-2.07** | **0.32** | **<0.001** |
| *R*^2^ | 0.23 |  |  | 0.23 |  |  | 0.26 |  |  |
| Number of individuals | 6,905 |  |  | 6,905 |  |  | 6,905 |  |  |
| Number of countries | 10 |  |  | 10 |  |  | 10 |  |  |

SE=Cluster-robust standard error. Estimates in bold represent p-values<0.05. Country dummy variables included.

Supplementary Figure S1. Estimation results of multilevel ordered logit regression.

Values of estimated average marginal effects (category “strongly agree”) are shown together with their 95% confidence intervals.

Supplementary Figure S2. Estimation results of Model 2, Table 3, without international students.

Values of estimated coefficients are shown, together with their 95% confidence intervals.

| **(a) Gender**  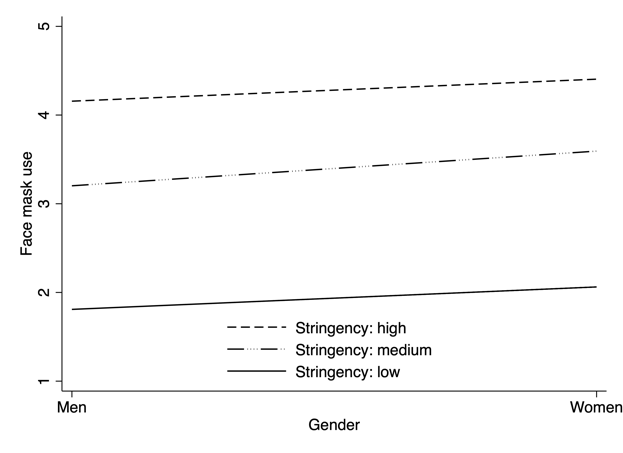 | **(b) Age**  **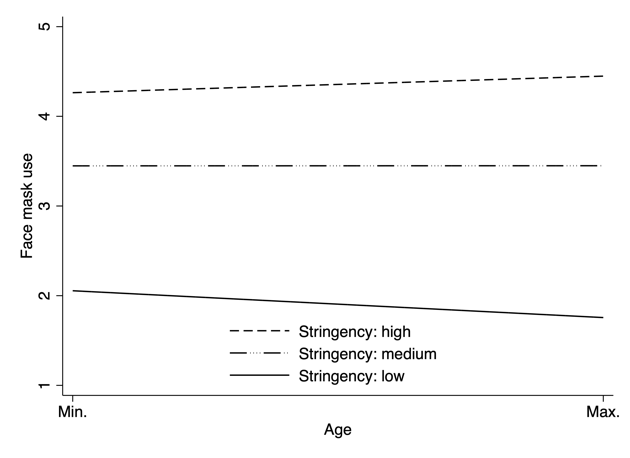** |
| --- | --- |
| **(c) Self-related risk perception COVID-19**   | **(d) Other-related risk perception COVID-19**   |
| **(e) Direct experience COVID-19**  **** | **(f) Indirect experience COVID-19**   |

Supplementary Figure S3. Interaction Plots Individual-Level Variables.
